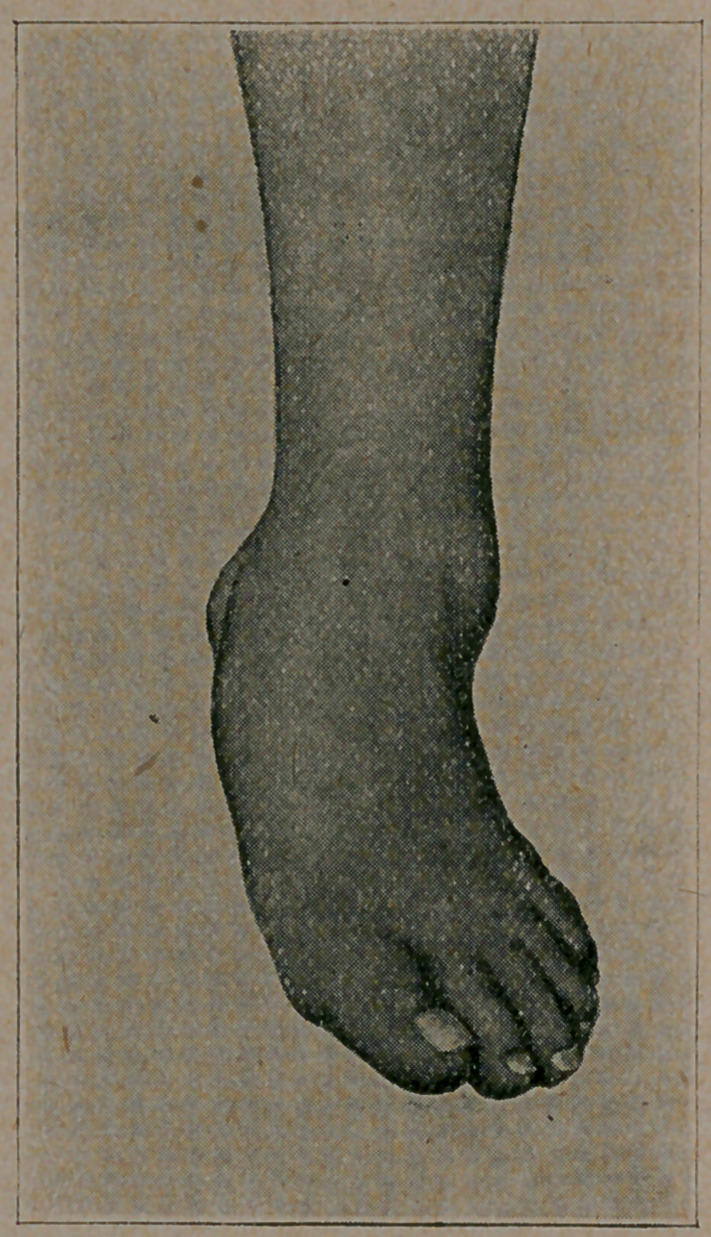# New York Academy of Medicine—Section in Orthopedic Surgery

**Published:** 1898-11

**Authors:** 


### Society Notes.

### New York Academy of Medicine—Section in Orthopedic Surgery.—Meeting of October 21, 1898.

### OBSCURE INJURY OF THE HIP.

Dr. G. R. Elliott presented a boy 2 years and 8 months old, who had fallen from a tree two months before. He complained of the left knee, but was able to walk and run. His father reported that the left foot had been dragged with a decided limp and everted to a right angle, and that its normal position had been restored after manual traction and manipulation. A slight limp had, however, persisted. The left leg was three-eighths of an inch short, and the left thigh one-half inch atrophied. Gentle manipulation seemed to produce a slight slipping of the joint. The child's ligaments were generally relaxed. He suggested the diagnosis of a dislocated hip reduced at once by manipulation.

Dr. N. M. Shaffer said that the limbs were practically of the same length, and that whatever might have been the lesion there were at this stage no positive signs of hip-disease, dislocation or separation of the epiphysis.

Dr. A. B. Judson found the trochanter enough above the line to make it probable that there had been a separation of the epiphysis.

Dr. T. H. Myers said that the limp might be from habit acquired when the hip was painful. The slight shortening in itself would not cause a limp. Irregularity in the length of the limbs had been said to be the rule rather than the exception. The cause of the shortening was not apparent since a dislocation, when reduced, should not leave any shortening.

Dr. R. H. Sayre had noticed the presence of marked knock-knee, and the father had said that the child had always turned in his toes. In other words, he had been unconsciously walking Indian fashion to make his feet more comfortable and to protect the arch of the foot. Beyond this the child appeared to be well.

Dr. P. J. Fiske thought that there might have been a bending of the femoral neck due to the accident, or acquired in some other way.

Dr. Elliott said that the head of the bone was in its socket, wherever it might have been immediately after the accident. He thought that the question of separation of the epiphysis remained undecided. He stated that the child had ridden a bicycle frequently since he was taught by his father to ride, when he was 18 months old. His greatest distance had been four miles. The boy was 36½ inches in height and his weight was 31 pounds. His bicycle weighed 11 pounds, diameter of wheel 13½ inches, crank 4 inches, wheel-base 21½ inches, gear 46 inches. He had ridden without trouble since the accident, but the exercise was at once forbidden when the patient was first seen, a few days ago. His brother, 4½ years of age, began to ride a wheel when 3 years old. He had a record of a 20-mile run, and was in perfect health.

THE USE OF THE BICYCLE BY CHILDREN.

Dr. Myers said that in the case of a child who rode a bicycle great care should be used in the adjustment of the height of the seat and the handle bar.

Dr. Sayre examined the boy's bicycle, and said that the construction of the seat was such that it would compel the patient to appear before the Section on Genito-Urinary Diseases later on. He did not see why a boy of that age should not ride a wheel if he kept off the street. The exercise should not be more than he could stand. Small children sometimes rode ponies and seemed to get along perfectly well.

Dr. Judson said that young children rode tricycles without attracting any especial attention. The bicycle furnished ischiatic support. In appropriate cases he advised its use when it was desirable to combine speedy and agreeable locomotion with relief of the lower extremities from carrying the weight of the body, and from the pressure and concussion incident to walking and running. The same was true of horse-back riding. Aside from the risk of accident, he thought that the moderate use of the bicycle at any age would promote normal development and health.

Dr. R. Whitman thought bicycle riding was a good exercise for knock-knees and weak feet.

Dr. H. L. Taylor strongly disapproved of bicycle riding for young children, not from an orthopædic standpoint, but on the ground of its being injurious to the general health.

Dr. Elliott said that children generally assumed bad attitudes on the wheel, leading to faulty development of the thorax. At an early age the bones were soft and the ligaments undeveloped and unfitted to stand the special requirements of riding a bicycle, and the result might be, as in the case of the patient, a relaxed ligamentous system. Bicycle riding by children tended to disproportionate development of the legs when compared with the arms. It should not take the place of general exercise, which developed the whole body alike.

TRAUMATIC SPINE.

Dr. Fiske exhibited a man 34 years of age, who had recovered from injury of the spine with paraplegia and rectal and vesical symptoms. The patient had been presented at the meeting of May 21, 1897.

There had been no return of the symptoms, and the recovery was now, more than four years after the accident, complete. The violence had been extreme, followed by rigidity and pain in the dorso-lumbar region, complete paralysis from the waist down and incontinence of fæces and urine. There had been no crepitus and no deformity. The patient was perfectly helpless. The diagnosis was severe spinal trauma, concussion of the cord, damage to ligamentous structures and probably partial dislocation with spontaneous reduction. Treatment had been by a plaster of paris jacket worn with occasional renewals for ten months. There had been no bedsores. Recovery with control of sphincters had been complete and the man was apparently in perfect health. In answer to questions, Dr. Fiske said that ankle clonus had not been present, that the lower part of the abdomen had been sensitive, but the scrotum, penis and sacrum were anæsthetic, that the sensory paralysis disapeared first, that there had been considerable atrophy of the muscles of the thigh and calf, probably from disuse, that the patient had felt nothing give way as he was immediately unsconcious, and that he began to use his legs in about four months, and could walk at the end of seven months. The anæsthesia of the scrotum and penis had led to the opinion that the injury was at the 18th dorsal vertebra and 1st lumbar.

Dr. Elliott thought that the lesion had not been above the first lumbar. Above that point, which was the end of the cord, there would probably have been destruction of the anterior horn cells, with ankle clonus and great localized atrophy. He could hardly conceive of anything less than this happening at a higher level after an injury attended with so much paralysis.

Dr. Shaffer had seen several such cases. The lower the point of injury the better would be the prognosis. The result had certainly been very good in this case, where there must have been a partial dislocation or fracture. He recalled the case of a man who was thrown from a vehicle and struck the ground in a sitting position. Rigidity of the spine had developed, but recovery had followed with perfect motion of the spine. A certain amount of compression of the anterior column could occur without serious results. If the posterior columns were injured, we would get symptoms such as had been present in the patient exhibited.

Dr. Sayre had seen a case similar to the one under consideration. In a railroad accident in which an express car had rolled down a bank, a man had been struck violently by the safe. He was paralyzed from the waist down with no control of the rectum or bladder. This condition lasted some three years. He gradually improved under treatment similar to that described, and had been restored to perfect health.

### FRACTURE OF THE SPINE

Dr. Whitman presented a patient with a rather different history. He was a young man 22 years of age, who had fallen twenty-five feet from a cliff. He could walk with assistance and, although he had pain, stiffness and weakness in the back, numbness and weakness in the legs, and pain in the lower part of the abdomen and the anterior surface of the thighs, he resumed work as a clerk at the end of a week. Dr. Whitman had examined him on August 8th, about two weeks after the accident, on account of a "lump," composed of the projecting spines of the second, third and fourth lumbar vertebræ. There was some pain on extensive motion of the back, and moderate rigidity at the seat of the fracture. A brace relieved the symptoms in a great degree, and at the end of a month he considered himself well, although he was still wearing the brace. It was seen that the normal lumbar lordosis had been replaced by a projection. Motion was practically normal. There had been fracture and compression of the vertebral bodies, and yet the symptoms had been insignificant.

Dr. Myers recalled the case of a man who had fractured his spine in a fall of twenty-five feet in a doubled forward position. Pain was not severe, but weakness in the lumbar region, the seat of the fracture, prevented sitting up or standing. He was in bed for three weeks and then walked with a cane. A kyphos was found and a spinal brace relieved his symptoms very quickly. He was well in six months. Fractures of the vertebræ often gave symptoms but poorly marked when compared with fractures in other locations. The most common symptom was weakness. Crepitus and false points of motion were not usually detected. Pain was moderate and deformity was frequently absent until after the patient had assumed the erect position for several days.

UNUSUAL FRACTURES OF THE NECK OF THE FEMUR.

Dr. Taylor presented a boy 15 years of age, who, in October, 1896, felt sudden severe pain in the right leg followed by lameness for two weeks. No shortening was noticed. After that he had lameness and disability with but little pain till January 3rd, 1897, when he slipped and fell on the floor with the knee bent under him. He was unable to rise or walk, and the neck of the right femur was found to be broken. He was treated by a plaster of paris application, and in July, 1897, when first seen by Dr. Taylor, he was limping badly, the trochanter was one inch above the line, there was extreme eversion and very limited motion. Crutches were advised. In December, 1897, the patient had been free from pain for many months, and there was increased motion. In April, 1898, under an anæsthetic, more mobility and lessened eversion were gained by manipulation, which was repeated in September, 1898, with further improvement.

Present condition of patient: Thirty degrees of free lateral motion, considerable free rotation and thirty degrees of flexion. Trochanter a full inch above the line. Walking was very free, but with a slight limp. An apparatus, soon to be laid aside, was worn to prevent outward rotation.

Dr. Taylor also presented a boy of 18 years, who, in December, 1897, fell on his left knee. There was immediate stinging pain in the left hip, but he could walk with some assistance. He soon walked with a cane, and three weeks after the fall there was a marked limp with very little motion in the hip. The limb was one inch short and rotated outward. The trochanter was one inch above the line, and there were tenderness, induration and muscular spasm about the hip. Treatment was by traction splint, long crutches and a high sole on the foot of the well side. In May, 1898, the patient had been free from pain for two or three months, and there was more motion. The splint was removed. In September a cane was substituted for the crutches.

Present status: Walking with considerable limp. No pain. Can raise the leg while lying. Shortening of one and one-half inches. Limited motion at the hip and adduction. These cases were of especial interest on account of the youth of the patients and the slight violence of the accidents.

Dr. Whitman said that the first patient doubtless had coxa vara, which weakened the neck of the femur, causing it to break under a moderate degree of violence. In three cases of coxa vara in young subjects he had operated by removing a wedge from the base of the trochanter in order to restore the neck to its normal position and strength. The second patient also probably belonged to the same class. He recalled the case of a young colored girl who, after a period of slight limping and outward rotation with slight stiffness of the hip and pain in the thigh, suffered a fall on her way to school. She was carried home with typical fracture of the neck of the femur. She was treated by the use of a traction splint with a favorable result.

Dr. Taylor said that he was confirmed in his opinion that bending of the neck of the femur had preceded the accident and had made easy the fracture of the bone in the case of the first patient presented. In the second case, however, there had been no previous signs or symptoms of deformity of the femoral neck, and such a condition must be considered hypothetical.

### CONGENITAL DISLOCATION OF THE HIP.

Dr. Elliott exhibited a further dissection of the specimen shown at the last meeting of the Section. (See The TEXAS MEDICAL JOURNAL, August, 1898, p. 74.—Editor.) The patient had been a girl 7 years of age. The dislocation of the right hip had been upward and forward. The neck had been found to be short and the muscles shortened and somewhat atrophied. During life there had been more than one inch of shortening, and the child had walked with difficulty, like one with weak muscles. The head had made a deep and extremely well defined acetabulum lined with cartilage, below and near the anterior superior iliac spine. The original acetabulum was almost equally well defined, measuring one and one-eighth inches in its vertical, and one inch in its transverse diameter with a depth of one-fourth inch. So well defined a first acetabulum at this age was rare. Lorenz cited one at the age of 18 years, and the older anatomists found them at very late periods of life. As a rule, however, the acetabulum not in use failed to keep pace with the development of the other parts, and at an age much younger than that of the specimen it was usual to find it rudimentary and frequently presenting a convex contour. The old acetabulum was found to contain some fat, but was chiefly occupied by an exceptionally large ligamentum teres, measuring one and one-half inches in length, three-fouths of an inch in width and three-sixteenths of an inch in thickness, running from a well defined cotyloid notch through the vertical diameter of the acetabulum to an insertion in the femoral head. As a rule the ligamentum teres had been found at the age of 3 or 4 years to be a mere ribbon, or to have disappeared. In the usual dislocation on the dorsum ilii, the disappearance of the ligament might be explained by the facts that it had no function and was compressed closely between the margin of the acetabulum and the femur. In the specimen, however, the displacement had been directly upwards, and the tremendous size of the ligament was apparently the result of its being called on to sustain the weight of the trunk at every step in walking. Its great size, then, was physiological rather than pathological.

Dr. Whitman said that the old acetabulum appeared to be of fair size, and that, as the tissues were doubtless far more yielding in life than in the preserved specimen, an operation by the open method, in which the hypertrophied ligament would have been removed, might have been successful.

Dr. Sayre said that, as the head was as broad as, if not broader than, the place where the acetabulum should be, it was doubtful whether chiseling away a part of the head would not have been required before reduction.

### TABETIC TALIPES VALGUS.

Dr. Judson presented a photograph of talipes valgus of the left foot in a man about 35 years of age, affected with locomotor ataxia of several years duration. It was an instance of Charcot's joint affecting the tarsus. The patient's right knee joint had been exsected for this condition, but stability had not been restored to the knee by the operation. Pathologically there were pulpy and fluid degeneration of the bony and other tissues and disintegration of the structures of the joints. Equino-varus also occurred in locomotor ataxia and in Friedreich's disease, but was the result not of bony changes but of abnormal muscular action. The primary disease was so serious and disabling that the question of treating these secondary affections was not often a practical one. Mechanical treatment might, however, be considered with three objects in view:

1, To give firmness to the foot and ankle and direct the sole to the ground; 2, to give lateral support to a Charcot's knee; and 3, to stiffen the knees by the use of automatic joints in order to prolong the period when locomotion is possible with the aid of crutches.

## Figures and Tables

**Figure f1:**